# The Secret Role of microRNAs in Cancer Stem Cell Development and Potential Therapy: A Notch-Pathway Approach

**DOI:** 10.3389/fonc.2014.00389

**Published:** 2015-02-11

**Authors:** Marianna Prokopi, Christina A. Kousparou, Agamemnon A. Epenetos

**Affiliations:** ^1^The Bank of Cyprus Oncology Centre, Trojantec Ltd., Nicosia, Cyprus; ^2^Department of Mechanical Engineering and Materials Science and Engineering, Cyprus University of Technology, Limassol, Cyprus; ^3^Imperial College London, London, UK; ^4^Clinical Oncology, The Harley Street Oncology Clinic, London, UK; ^5^Medical Oncology, St Bartholomew’s Hospital, London, UK

**Keywords:** Notch signaling, cancer stem cells, microRNAs, cancer therapeutics, miRNA therapeutics

## Abstract

MicroRNAs (miRNAs) have been implicated in the development of some if not all cancer types and have been identified as attractive targets for prognosis, diagnosis, and therapy of the disease. miRNAs are a class of small non-coding RNAs (20–22 nt in length) that bind imperfectly to the 3′-untranslated region of target mRNA regulating gene expression. Aberrantly expressed miRNAs in cancer, sometimes known as oncomiRNAs, have been shown to play a major role in oncogenesis, metastasis, and drug resistance. Amplification of oncomiRNAs during cancer development correlates with the silencing of tumor suppressor genes; on the other hand, down-regulation of miRNAs has also been observed in cancer and cancer stem cells (CSCs). In both cases, miRNA regulation is inversely correlated with cancer progression. Growing evidence indicates that miRNAs are also involved in the metastatic process by either suppressing or promoting metastasis-related genes leading to the reduction or activation of cancer cell migration and invasion processes. In particular, circulating miRNAs (vesicle-encapsulated or non-encapsulated) have significant effects on tumorigenesis: membrane-particles, apoptotic bodies, and exosomes have been described as providers of a cell-to-cell communication system transporting oncogenic miRNAs from tumors to neighboring cells and distant metastatic sites. It is hypothesized that miRNAs control cancer development in a traditional manner, by regulating signaling pathways and factors. In addition, recent developments indicate a non-conventional mechanism of cancer regulation by stem cell reprograming via a regulatory network consisting of miRNAs and Wnt/β-catenin, Notch, and Hedgehog signaling pathways, all of which are involved in controlling stem cell functions of CSCs. In this review, we focus on the role of miRNAs in the Notch-pathway and how they regulate CSC self-renewal, differentiation and tumorigenesis by direct/indirect targeting of the Notch-pathway.

## Background

Cancer is a heterogeneous disease with cellular hierarchies and many different phenotypes. Strong evidence points to the fact that the majority of cells in solid tumors are of non-tumorigenic origin. A small population of progenitor cells is thought to be responsible for tumor initiation, growth, metastasis, drug resistance, and recurrence. These cells are known as cancer stem cells (CSCs), since they possess stem cell characteristics such as self-renewal, asymmetric cell division, differentiation, and chemoresistance (1, 2). CSCs act via signaling pathways that mediate self-renewal, including Notch and Wnt. It is becoming widely accepted that irregular stem cell self-renewal is essential for cancer initiation, formation, and relapse and that CSCs play a central role in cancer cell biology. Thus the identification of specific markers of CSCs may be important in the discovery and development of novel oncology therapeutics.

## Notch Signaling Pathway

The authors support the hypothesis that cancer initiation and development involve the improper activation of developmental signaling pathways. Normally such pathways control the growth of tissues and organs by maintaining the balance between cell proliferation, differentiation, senescence, and apoptosis. Notch signaling pathway plays a key role in stem cell self-renewal, cell proliferation, and differentiation. Consequently, it has important developmental functions, and its aberrant activation leads to many diseases and cancers (3–5). *Notch* genes encode large single pass transmembrane proteins that regulate cell fate determination (6). Previous studies in Drosophila, *Caenorhabditis elegans*, and mammalian cell cultures have shown that Notch act as receptors for the DSL (Delta, Serrate, and Lag-2) family of ligands and signal through two downstream pathways. One of these is via the CSL (CBF1, Suppressor of Hairless, Lag-1) family of transcription factors and the other via the cytoplasmic adapter protein Deltex. In mammals, the Notch signaling pathway includes four receptors (Notch 1–4) and five ligands (Delta-like 1, 3, and 4 & Jagged-1 and 2) (7). Notch signaling is initiated through ligand–receptor binding between two neighboring cells. Upon activation, Notch undergoes cleavage, releasing its intracellular domain NICD and translocates into the nucleus for transcriptional activation of its downstream target genes (8).

## Notch in Tumorigenesis Mediated by CSCs

Modifications in the Notch-pathway and its associated genes can result in ligand dysregulation having dramatic developmental effects in humans, thus implicating Notch signaling in several inherited diseases such as Cerebral Autosomal Dominant Arteriopathy with Subcortical Infarcts and Leukoencephalopathy, Alagille syndrome, and Spondylocostal dysostosis. Since the discovery of *Notch1* gene alterations in T-cell acute lymphoblastic leukemia/lymphoma, deregulated Notch signaling has been connected to many solid tumor pathologies and different cancer types (leukemia, neuroblastomas, skin, cervical, lung, prostate, and breast cancer) (4, 9, 10). The role of Notch signaling in tumorigenesis is thought to be mainly oncogenic, although some observations have suggested an anti-proliferative role in a small number of cancers (hepatocellular carcinoma and skin cancer) (11–13). The oncogenic function of Notch signaling is associated with high levels of Jagged-1, mainly in prostate and breast cancer, as well as with loss of Numb activity – a negative regulator of Notch-pathway – in 40% of breast cancers and 30% of lung cancers (5, 14, 15). Thus, the deregulation of the Notch signaling pathway has so far been linked to metastasis, recurrence, and reduced overall survival. On the other hand, tumor aggressiveness has been linked to the cross-talk between Notch and other oncogenic pathways such as Wnt/β-catenin, NF-kB, Ras, and Akt (16–18).

Although irregular activation of a single pathway may result in tumorigenesis, oncogenic pathways rarely operate in isolation. Cross-talk between signaling pathways adds to the complexity of the disease and are heavily influenced by the microenvironment. Recent studies have revealed that the interaction between the Notch and Wnt/β-catenin signaling pathways drives the CSC uncontrolled self-renewal, resulting in CSC-related tumor recurrence after treatment (19, 20). Notch signaling regulates both the CSC formation and the epithelial-to-mesenchymal-transition (EMT) phenotype during tumor progression. The EMT process, which occurs during tumor progression, drives the CSCs to become metastatic. Indeed, Notch-mediated EMT converts polarized epithelial cells into motile, invasive cells due to loss of E-cadherin – a membrane glycoprotein involved in the adherence of adjacent cells – which results in β-catenin activation and dissemination of cancer cells and CSCs from the primary tumor (21–23). Notch signaling pathway interacts with several oncogenic pathways, transcription, and growth factors (e.g., Snail, Slug, and TGF-β) regulating various biological and pathologic processes during cancer development, progression, and therapy. However, a growing body of evidence indicates that Notch is regulated at molecular level via cross-talk with miRNAs suggesting a critical role for these molecules in tumor biology (24, 25).

## MiRNA Biogenesis

MiRNAs are a class of small, non-coding RNAs that regulate mRNA by acting at the post-translational level (26). The interaction between miRNAs and mRNAs is highly complex; in particular each miRNA can control hundreds of gene targets underlining the extraordinary impact of miRNA on protein expression. We are just beginning to understand how this novel class of regulators affects processes, at least, in mammals. Processed from longer primary transcripts by Drosha and Dicer, miRNAs bind through imperfect complementarity to their target genes at the seed sequence (eight-base long), of the 3′ non-coding region leading to degradation of target mRNA due to deadenylation/mRNA cleavage or to repression of mRNA translation initiation (27, 28). MiRNAs have been implicated in a wide range of cell functions – normal or pathological – comprising of cell proliferation, apoptosis, differentiation, and self-renewal (2, 29). Therefore, dysregulation of miRNAs is linked to a range of human pathologies including cancer and its expression is associated with cancer development, progression, and prognosis mainly because of their involvement in cell proliferation and apoptosis (30). MiRNAs were shown to be differentially expressed in cancer forming unique miRNA patterns with some miRNAs to have oncogenic activity while others have tumor suppressor activity (oncosuppressors). Thus, oncogenic miRNAs are upregulated in cancer whereas tumor suppressor miRNAs are downregulated (31). With regards to cancer biology, miRNAs targeting oncogenes are often located in fragile regions with a tension to be downregulated in tumors leading to overexpression of their target oncogenes. A breakthrough study showed that 50% of the annotated human miRNAs are positioned in these unstable sites and are associated with cancer and function in tumor progression (32).

## Cancer and miRNAs

Tissue-specific/tissue-enriched miRNAs, often deregulated, play a major role in cancer progress functioning as both oncosuppressors and oncogenes. For example, upregulated miR-21 is associated with breast and lung cancer, glioblastoma, leukemia, neuroblastoma, and liver metastasis (33, 34). Brain-specific neuromiR-124 is downregulated in glioblastoma resulting in increased CSC numbers and oncogenic capacity (35). On the other hand, let-7, normally expressed in lung, is downregulated in lung cancer and associated with poor survival (36), lung-specific pneumomiR-29 suppresses tumorigenicity in non-small cell lung cancer cells and miR-143 and miR-145 have been shown to be downregulated in breast, cervical, and colorectal cancers (37, 38). Moreover, loss of miR-15 and miR-16 has been related to chronic lymphoid leukemia by negatively regulating the anti-apoptotic gene *BCL2*, supporting a role in the immune system (39, 40). Similarly, many other miRNAs have been related to Notch signaling pathway, by regulating Notch-associated genes and affecting many types of cancer (Table 1).

**Table 1:** **Important miRNAs regulating Notch signaling pathway and Notch-associated genes**.

miRNA	Cancer type/cancer cells	Notch-related target
miR-34 family	Pancreatic CSCs	Bcl-2/Notch (42, 46)
miR-34a	Pancreas, melanoma, lung, breast, and glioma	p53/Notch1/Notch2/Jagged-1/Hes-1 (24, 57)
miR-34a	Prostate and colon CSCs	Notch1 (47, 58)
miR-34b/c	Melanoma and glioma	Notch1/Notch2 (45, 59)
miR-34c-3p	Glioma	Notch2 (45)
miR-200 family	Prostate	Jagged-1/Notch (49)
miR-200	Squamous esophangeal	ZEB-1/Notch3 (51)
miR-200b	Pancreatic cancer cell line	Jagged-1/2, Hes-1, Heg2, Bcl-2 (50)
miR-200c/miR-141	Pancreatic adenocarcinoma and basal type of breast cancer	ZEB-1/Notch (Jagged-1, Maml2, Maml3) (48)
miR-199-5p	Medulloblastoma and osteosarcoma	Notch1/Jagged-1/Hes-1/Dll-1 (25)
miR-146a	Breast	Notch/Numb (53)
miR-1	Colorectal	NOTCH3 (55)
miR-143	Glomus tumors	NOTCH1-3 (56)

## Important miRNAs Controlling Cancer via Regulation of Notch Signaling Pathway

### MiR-34 Family

**Figure 1:**
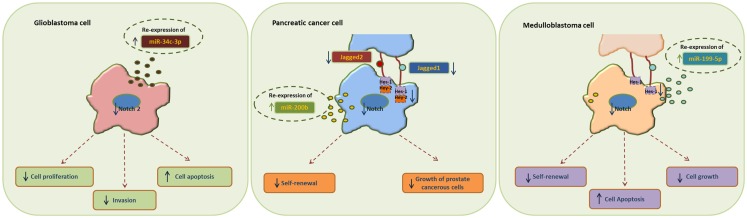
**Examples of miRNA re-expression therapy using miR-34, miR-200, and miR-199 family, in glioblastomas, pancreatic, and medulloblastomas cancer cells, all affecting the Notch signaling pathway**.

MiR-34 family is composed of miR-34a, miR-34b, and miR-34c and has been connected to the regulation of p53 and Notch signaling pathway. Low levels or no expression of miR-34 corresponds to higher probability of breast, brain, pancreatic, and non-small cell lung cancer suggesting a tumor suppressor role (24, 41, 42). Repression of miR-34a was identified in a panel of tumor-derived cancer cell lines such as pancreas, melanoma, lung, breast, and glioma (43, 44). However, miR-34b/c have been identified in malignant melanoma cases and glioma patients’ tissues (45). The involvement of miR-34 family has now been shown to affect major properties of CSCs. For example, it has been revealed that restoration of miR-34 inhibits CD44^+^/CD133^+^ pancreatic CSCs self-renewal capacity through direct down-regulation of Bcl-2 and Notch signaling pathways (46). Furthermore, transfection with miR-34c-3p in U251 and U87 glioblastoma cell lines inhibited cell proliferation, induced cell apoptosis, and obstructed glioma cell invasion. Therefore, it was established that miR-34c-3p overexpression reduced the levels of Notch 2 (Figure 1) indicating that cell proliferation inhibition occurred via the Notch signaling pathway (45). On the other hand, it is well recognized that Notch 1 is linked to CSCs “stemness” characteristics and promotes the EMT phenotype, which is closely linked to many types of metastatic cancer. In prostate cancer for instance, upregulated levels of Notch 1 are highly associated with prostate cancer development, metastasis, and progression. Re-expression of miR-34a in C4-2B and CWR22rv1 prostate cancer cells reduced the expression of Notch1, decreased the self-renewal capacity and inhibited the growth of prostate cancer cells (47). Taken together, miR-34 family directs the regulation of Notch 1 and 2 protein expression in glioma cells, pancreatic, and prostate cancer cells mediating the suppression of self-renewal and differentiation properties of CSCs. Restoration of miR-34 levels could be eventually used as cancer therapeutic by down-regulating the Notch family members.

### MiR-200 Family

MiR-200 family consists of five members: miR-200a, miR-200b, miR-200c, miR-141, and miR-429. Recent studies have shown that the miR-200 family is highly implicated in the regulation of CSCs with examples involving breast, colorectal, prostate, and brain CSCs. Low levels of miR-200c and miR-141 are correlated with high expression of ZEB-1 – an EMT activator – which in turn activates Notch signaling pathway by targeting the Notch ligand Jagged-1 and Notch co-activators Maml 2 and 3. Reduced expression of miR-141 and miR-200c affects stem cell properties and drug resistance in two human cancer types’ pancreatic adenocarcinoma and basal type of breast cancer (48). Moreover, re-expression of miR-200 family including miR-141 and miR-429 directly inhibits Jagged-1 in human metastatic prostate cancer cells suggesting a new way to control the fate of Notch-pathway (49). Similarly, in another study transfection of miR-200b in Rink-1 cells (pancreatic cell line) have reduced the levels of Jagged-1/2 and these of their target genes Hes-1, Hey-2 (Figure 1), and Bcl-2 leading to cell growth inhibition (50). Another critical study connects NOTCH3 in the regulation of ZEBs and the miR-200 family revealing how critical is Notch in EMT, invasion and tumor formation in squamous esophageal cancers (51). However, more in-depth investigation is required in order to understand how the miR-200 family regulates the Notch signaling pathway.

### MiR-199 Family

MiR-199-5p has been linked to the transcription factor Hes-1 in medulloblastoma tumors where it regulates cell growth and several CSC genes via Notch signaling pathway. In metastatic cancer patients’ miR-199-5p expression is lost, yet re-expression of it blocks the Notch signaling pathway (Figure 1) and the population of medulloblastoma stem cell like cells is decreased (25). Zollo’s group went a step further by developing stable nucleic and lipid particles (SNALPs) to deliver miR-199-5p in different tumorigenic cell lines such as colon, breast, glioblastoma, and medulloblastoma. Impairment of cell proliferation and CSC-associated markers was due to effect of miR-199-5p delivery via SNALPs however, different efficacies due to cell type cannot be excluded and the efficiency of these carriers can be limited by their instability and non-specific targeting *in vivo* (52).

### Single miRNAs regulating Notch

Recently, miR-146a was also found to interact with Notch via regulation of Numb in breast carcinomas (53) and to stimulate NF-κB activity through Notch 1 [reviewed in Ref. (54)]. Another miRNA involved in Notch-pathway is miR-1, which it was reported that directly regulates Notch via Dll-1 protein in mouse embryonic stem cells. MiR-1 has been linked to human hepatocellular carcinoma, lung, prostate, and head and neck cancers; therefore it is only a matter of time for Notch to be associated with the above through miR-1 [reviewed in Ref.(54)]. A recent study by Furukawa (55) demonstrated the association of miR-1 in colorectal tumors and the potential to suppress NOTCH3 expression, which in turn results in reduction of *Asef* regulating the growth, migration, and invasion potential of cancer cells. Other reports, show an association of *MIR143* gene with *NOTCH1-3*; suggesting that the mechanism of *MIR143*–*NOTCH1-3* tumorigenesis is through oncogenic activation of NOTCH driven by the very strong *MIR143* promoter in malignant glomus tumors (56).

### Therapeutic applications of miRNAs in cancer

The development of therapies against cancer and CSCs has driven toward a new generation of cancer therapeutics. RNA-based approaches promise to be one of the next major classes of cancer therapeutics. Advancements in genetics relating to the role of RNA in an ever-expanding range of cellular pathways and processes have shown that RNA has many of the genetic and regulatory properties formerly attributed only to DNA and proteins. Current work is based on modified mRNA regulating the expression of therapeutic proteins and on RNAi variants such as siRNA and miRNA, which can either block oncogenes or amplify oncosuppressor genes in cancer cells. The success of RNAi therapeutics hinges on their effective and safe delivery to their molecular targets inside cancer cells and tumors. However, targeted delivery remains a major challenge in miRNA therapy because naked ribonucleic acids are subject to rapid degradation by serum nucleases and miRNAs cannot diffuse freely into cells. To deliver the new class of targeted drugs, several technologies are under development including nanocapsules and nanocarriers, micro/nanoparticles, liposomes, and PEGylated vesicles. Delivery of miRNAs or miRNA inhibitors requires a flexible and efficient delivery system, for example, miR-34a has being encapsulated in stable-acid-lipid particles used to target Dll-1 *in vitro* (52). Other delivery approaches involve the use of agents such as polyethylenimine (PU-PEI) to mediate miRNA delivery. PU-PEI-mediated miR-145 delivery vehicle has been used successfully for miR-145 delivery to glioblastoma cells (60). Another candidate for the successful miRNA therapeutic approach is the vector-mediated overexpression of miRNAs to tumor tissues *in vivo* using adenoviral or lentiviral delivery. Of note, miR-26a expression in normal liver and liver tumor cells succeeded via adenoviral delivery in order to inhibit tumor cell proliferation and to lead in apoptosis (61). However, the use of this approach can only be employed in systemic local delivery. As the early clinical trial failures in the field have demonstrated, delivery of RNAi therapeutics to their molecular targets inside cancer cells is crucial. The authors have developed a novel system for miRNA delivery applicable for both local and systemic administration with the use of mesenchymal stem cell (MSC)-derived microparticles (MPs). The idea was based on the fact that miRNAs are released in the blood-stream via a controlled and active process through MPs, exosomes, and apoptotic bodies. MiRNAs associated with cellular particles were found to be resistant from nuclease degradation and able to be transferred in a variety of cells and alter the gene expression of the recipient cells (62–64). The novel technology designs for MSC-derived MPs programed to enclose and deliver specific miRNAs that affect the action of genes associated with cancer growth, neovascularization, and metastasis (65). MSC-derived MPs retain the membrane receptors that allow MSCs to home selectively into tumor sites and target malignant cells, thus avoiding the targeting of healthy cells. MSC-derived MPs home and engraft in solid tumors via specific chemokine receptors, fuse to tumor cell membranes, and incorporate miRNA directly into the target cancer cell, thus exerting their therapeutic effect while minimizing side effects associated with conventional therapies (65, 66). MSC-derived MPs offer enhanced therapeutic potential due to their ability to target multiple molecules in malignant cells when compared with approaches targeting single genes and induce immunosuppression through cytokine signaling inhibition making this approach useful in therapy of recurring cancer disease (65, 67).

## Concluding Remarks

As miRNA expression seems to be altered in many human diseases, including cancer, the miRNA revolution has already begun and has set the stage for “miRNA re-expression therapy.” Among the many genes that miRNAs can regulate are oncogenes and tumor suppressors, targets of drugs currently used in the clinic. Although a few miRNAs are overexpressed in cancer and seem to function as oncogenes themselves, a greater number of miRNAs have been shown to be downregulated in cancer and have the potential to act as tumor suppressors. MiRNA re-expression and down-regulation have both been shown to have antitumor effects while re-expressing a tumor suppressor miRNA could downregulate multiple oncogenes. Re-expression, to physiological levels, of tissue-specific miRNAs that are lost in cancer can induce the de-differentiation of cancer cells. Re-expressing lost miRNA in a cell can deliver a dramatic effect, because miRNAs regulate a vast number of genes and pathways. However, similar to other RNA-related therapies the key challenge remains the inadequate delivery and stability of the therapeutic agents to the tumor site. MiRNA antagonists and mimics are already available in the market but limited to local administration applicable to only a few target tissues. On the other hand, miRNA therapeutics could follow similar approaches to siRNA chemistry, vector-based systems as in gene therapy, and/or vehicle-based delivery systems. Understanding miRNA biology and how it contributes to cancer development it will provide for new diagnostic and therapeutic tools. MiRNA-based therapy is not a traditional approach and will need further development and improvements in composition, stability, and delivery to the target areas. Nevertheless, miRNA profiling will open a new era in cancer biology providing a new and improved cancer classification system.

## Conflict of Interest Statement

The Guest Associate Editor Aleksandra Filipovic declares that, despite having collaborated with author Agamemnon A. Epenetos, the review process was handled objectively and no conflict of interest exists. The authors declare that the research was conducted in the absence of any commercial or financial relationships that could be construed as a potential conflict of interest.
